# Recombinant *Toxoplasma gondii* phosphoglycerate mutase 2 confers protective immunity against toxoplasmosis in BALB/c mice

**DOI:** 10.1051/parasite/2016012

**Published:** 2016-03-16

**Authors:** Hai-Long Wang, Li-Min Wen, Yan-Jiang Pei, Fen Wang, Li-Tian Yin, Ji-Zhong Bai, Rui Guo, Chun-Fang Wang, Guo-Rong Yin

**Affiliations:** 1 Academy of Basic Medicine, Shanxi Medical University Taiyuan Shanxi 030001 PR China; 2 Department of Biochemistry and Molecular Biology, Shanxi Medical University Taiyuan Shanxi 030001 PR China; 3 Department of General Surgery, Xi’an Red Cross Hospital Xi’an Shanxi 710000 PR China; 4 Department of Infection Control, The Central Hospital of Enshi Prefecture Enshi Hubei 445000 PR China; 5 Department of Physiology, Faculty of Medical and Health Sciences, University of Auckland Private bag 92-019 Auckland 1142 New Zealand; 6 Laboratory Animal Center, Shanxi Medical University; Shanxi Key Laboratory of Laboratory Animals and Animal Models of Human Diseases Taiyuan Shanxi 030001 PR China

**Keywords:** *Toxoplasma gondii*, Phosphoglycerate mutase 2, Recombinant protein, Mucosal immunity, Nasal vaccine

## Abstract

Toxoplasmosis is one of the most widespread zoonoses worldwide. It has a high incidence and can result in severe disease in humans and livestock. Effective vaccines are needed to limit and prevent infection with *Toxoplasma gondii.* In this study, we evaluated the immuno-protective efficacy of a recombinant *Toxoplasma gondii* phosphoglycerate mutase 2 (rTgPGAM 2) against *T. gondii* infection in BALB/c mice. We report that the mice nasally immunised with rTgPGAM 2 displayed significantly higher levels of special IgG antibodies against rTgPGAM 2 (including IgG1, IgG2a and IgAs) and cytokines (including IFN-γ, IL-2 and IL-4) in their blood sera and supernatant of cultured spleen cells compared to those of control animals. In addition, an increased number of spleen lymphocytes and enhanced lymphocyte proliferative responses were observed in the rTgPGAM 2-immunised mice. After chronic infection and lethal challenge with the highly virulent *T. gondii* RH strain by oral gavage, the survival time of the rTgPGAM 2-immunised mice was longer (*P* < 0.01) and the survival rate (70%) was higher compared with the control mice (*P* < 0.01). The reduction rate of brain and liver tachyzoites in rTgPGAM 2-vaccinated mice reached approximately 57% and 69% compared with those of the control mice (*P* < 0.01). These results suggest that rTgPGAM 2 can generate protective immunity against *T. gondii* infection in BALB/c mice and may be a promising antigen in the further development of an effective vaccine against *T. gondii* infection.

## Abstract (fr)

La toxoplasmose est une des zoonoses les plus répandues dans le monde entier. Elle a une forte incidence et entraîne des dommages graves pour les humains et le bétail. Des vaccins efficaces sont nécessaires pour limiter et empêcher l’infection par *Toxoplasma gondii*. Dans cette étude, nous avons évalué l’efficacité immunitaire protectrice d’une phosphoglycérate mutase 2 recombinante de *T. gondii* (rTgPGAM 2) contre l’infection par *T. gondii* chez les souris BALB/c. Nous rapportons que les souris immunisées par voie nasale avec rTgPGAM 2 montraient des niveaux significativement plus élevés d’anticorps IgG spécifiques contre rTgPGAM 2 (y compris IgG1, IgG2a et IgA) et de cytokines (y compris IFN-γ, IL-2 et IL-4), dans leur sérum sanguin et dans le surnageant de cellules de rate en culture, par rapport à ceux des animaux témoins. En outre, un nombre accru de lymphocytes dans la rate et des réponses prolifératives lymphocytaires améliorées ont été observées chez les souris immunisées par rTgPGAM 2. Après l’infection chronique et une provocation létale avec la souche de *T. gondii* RH, très virulente, par gavage oral, le temps de survie des souris rTgPGAM 2 immunisées était plus long (*P* < 0.01) et le taux de survie (70 %) était plus élevé que chez les souris témoins (*P* < 0.01). Le taux de réduction des tachyzoïtes du cerveau et du foie chez les souris vaccinées par rTgPGAM 2 a atteint environ 57 % et 69 % par rapport à ceux des souris témoins (*P* < 0.01). Ces résultats suggèrent que rTgPGAM 2 peut générer une immunité protectrice contre une infection par *T. gondii* chez les souris BALB/c et peut être un antigène prometteur dans la poursuite du développement d’un vaccin efficace contre l’infection par *T. gondii*.

## Introduction

Toxoplasmosis is a disease caused by *Toxoplasma gondii*, which is an obligate intracellular parasite with a complex life cycle. *Toxoplasma gondii* is considered the most prevalent parasitic zoonotic pathogen worldwide [41] because up to 20–30% of the world’s population is infected [13]. Vaccination is a promising strategy for the prevention of *T. gondii* infection [44]. In recent years, significant progress has been made in the identification of vaccine candidates against toxoplasmosis [2, 17, 22, 51]. However, all existing vaccine candidates provide only partial protective efficacy against *T. gondii* infection [12, 52, 53]. Therefore, the identification of effective vaccine candidates against *T. gondii* would be of great value for the control of this parasitic infection in humans and animals. Phosphoglycerate mutase (PGAM) catalyses the reversible conversion of 2-phosphoglycerate (2-PG) to 3-phosphoglycerate (3-PG). This is an essential component of the glycolysis pathway providing 2-PG to the enzyme enolase, and in the gluconeogenesis pathway, where it supplies 3-PG to phosphoglycerate kinase [21]. There are two isoforms of PGAM in *T. gondii* (TgPGAM). TgPGAM 1 is proposed to be encoded by pseudogenes because the open reading frame (ORF) is not able to be amplified using repeated PCR cloning, and TgPGAM 2 is localised to the apicoplast and is involved in the glycolysis pathway [14]. In a *T. gondii* tachyzoite-infected host cell line model, TgPGAM 2 was upregulated in the infected cells compared with controls [1, 33]. In our previous study, TgPGAM 2 was identified as one component of soluble tachyzoite antigens [27]. Recently, TgPGAM was detected in excretory secretory antigens (ESA) prepared from RH strain tachyzoites administered to mice via intraperitoneal infection. Moreover, this PGAM can be recognised by anti-*Toxoplasma* IgM, IgG and IgA [36], which suggests TgPGAM may be a vaccine candidate.

The Tg*PGAM 2* gene was cloned from RH strain tachyzoites successfully, and recombinant TgPGAM 2 (rTgPGAM 2) was obtained and purified. The latter was demonstrated to be antigenic in our previous study [48]. In this study, rTgPGAM 2 was used for the immunisation of BALB/c mice, and systemic and mucosal immunity were examined in these immunised mice. After challenge with *T. gondii* RH strain tachyzoites, the numbers of tachyzoites in brains and livers were recorded. In addition, the survival time and survival rate were observed and measured in BALB/c mice immunised with rTgPGAM 2 and phosphate-buffered saline (PBS).

## Materials and methods

### Animals and parasites

Six-week-old female BALB/c mice were purchased from the Institute of Laboratory Animal Sciences, Chinese Academy of Medical Sciences. All mice were maintained under conventional, non-specific pathogen-free (non-SPF) conditions with 12 h light/dark cycles and free access to food and filtered water. *T. gondii* tachyzoites (RH strain) were kindly provided by the Peking University Health Science Centre and were maintained by serial intraperitoneal passaging in BALB/c mice. All experimental procedures were reviewed and approved by the Research Ethics Review Board at Shanxi Medical University under Protocol Number 20110320-1.

### Expression and purification of the recombinant protein

rTgPGAM 2 was expressed and purified as described previously [48]. Briefly, the ORF of the Tg*PGAM 2* gene was amplified from the RH strain *T. gondii* tachyzoites and cloned into the pET-30a(+) vector. The rTgPGAM 2 protein was induced to express in BL21 (DE3) cells at 37 °C with 0.2 mM IPTG and purified via Ni^2+^-NTA agarose (Qiagen, Germany) with 200 mM imidazole elution at 4 °C. Endotoxin was removed by using a ToxinEraser^TM^ Endotoxin Removal Kit and the endotoxin level was measured by the Chromogenic End-point Endotoxin Assay Kit (Horseshoe Crab Reagent Manufactory, Xiamen, China). Less than 0.1 EU/mL endotoxin was detected in the final protein preparations. Before inoculation into mice or stimulation *in vitro*, rTgPGAM 2 was dialysed against PBS, filtered through a 0.2-μm pore membrane and stored at −70 °C. The purified recombinant protein was quantified by the bicinchoninic acid (BCA) method.

### Immunisation and challenge

The mice were divided into five groups with 10 mice per group for assessing the immune responses evoked by rTgPGAM 2. They were intranasally immunised with 10, 20, 30 or 40 μg of rTgPGAM 2 that was suspended in 20 μL of sterile PBS, and PBS was used as a control. Each dose of immunogen (10 μL/nostril) was infused into the nostrils of mice with a micropipette. The mice were immunised using the same protocol on days 0, 14 and 21. Two weeks after the final inoculation (on day 35), the mice were anaesthetised with sodium pentobarbital (1.5%, 0.1 mL/20 g weight). Blood samples were collected from the mice through retro-orbital plexus puncture and were stored at −20 °C for analysis. Spleens were collected under aseptic conditions to perform lymphocyte proliferation assays, and the culture supernatants were used for cytokine assays.

To evaluate the protective efficacy of rTgPGAM 2 against *T. gondii* infection, on the 15th day after the last immunisation with PBS and 30 μg of rTgPGAM 2, eight mice in each group were challenged orally with 1 × 10^4^ *T. gondii* RH strain tachyzoites for the tachyzoite load assay, and 12 mice in each group were challenged orally with 4 × 10^4^ tachyzoites for the survival assay. The numbers of tachyzoites in the brains and livers of the mice were measured to assess the results of the chronic challenge infection assay. To assess the protective effect of rTgPGAM 2 in the infected mice, they were monitored at 8 am, 2 pm and 8 pm daily, and the time to death and survival were recorded and assessed for one month after parasite challenge.

### Preparation of mucosal washes

Murine nasal washes and vaginal secretions were prepared using previously described methods [6]. Small intestine washes were collected according to published protocols [46]. The suspensions were centrifuged at 200 × *g* for 15 min at 4 °C to remove any tissue, cellular debris and other impurities, and the supernatants were stored at −20 °C to assess the secretory immunoglobulin A (sIgA) levels.

### Antibody response by ELISA

The antigen-specific IgA, IgG, IgG1 and IgG2a antibodies in serum samples were evaluated by enzyme-linked immunosorbent assays (ELISAs) according to published protocols [45]. Briefly, 96-well flat-bottom microtiter plates were coated with 1 μg of rTgPGAM 2 in 100 μL sodium carbonate buffer (pH 9.2) overnight at 4 °C and then washed with PBS containing 0.05% Tween 20 (PBST). PBS containing 5% foetal calf serum (FCS) was used for blocking non-specific binding sites for 2 h at 37 °C. After washing three times with PBST, individual sera (100 μL/well) diluted in 1% bovine serum albumin (BSA)-PBST (1:200 for IgG, 1:50 for IgA, IgG1 and IgG2a) were transferred to the wells, followed by incubation for 1 h at 37 °C. The bound antibodies were detected by adding 50 μL of horseradish peroxidase-conjugated goat anti-mouse IgA, IgG, IgG1 or IgG2a (ProteinTech Group, Inc., USA) diluted 1:2000. The immune complexes were revealed by incubation with ortho-phenylenediamine (Sigma) and 0.15% H_2_O_2_ for 30 min, and the enzyme reaction was then terminated by adding 1 M H_2_SO_4_. The optical density (OD) was measured in an ELISA reader at 492 nm. All samples were examined in triplicate.

### Isolation and counting of intestinal intraepithelial lymphocytes and lymphocytes from Peyer’s patches

Intestinal intraepithelial lymphocytes (IELs) and lymphocytes from Peyer’s patches (PP) were isolated according to published protocols [26, 30]. Cells were filtered through nylon wool or 250-μm gauge steel mesh to remove undigested tissue pieces and the pellet debris. The suspended cells were counted in a haemocytometer to assess the number of IELs and PP lymphocytes.

### Spleen lymphocyte isolation, counting and proliferation *in vitro*

On the 15th day after the last immunisation, spleen lymphocytes were isolated and counted according to protocols [45]. Then, 2 × 10^5^ cells/well were cultured in 96-well plates in triplicate in RPMI-1640 containing 10% FCS and stimulated with 10 μg/mL of rTgPGAM 2, concanavalin A (Con A: 5 μg/mL, Sigma) or medium alone (negative control) for 72 h at 37 °C in 5% CO_2_. A cell counting Kit-8 (Dojindo Laboratories; Kumamoto, Japan) was used, and the results were expressed as the stimulation index (SI), which is the ratio of the OD_450_ of the stimulated cells to the OD_450_ of the unstimulated cells.

### Cytokine assays

Cytokines in serum and cell-free supernatants from cultured 1.5 × 10^6^ spleen lymphocytes stimulated with 10 μg of rTgPGAM 2 in 24-well plates were evaluated using a commercial ELISA kit (PeproTech, USA) according to the manufacturer’s instructions. Cell-free supernatants were harvested and assayed for IL-2 and IL-4 activities at 24 h, IL-10 activity at 72 h and gamma-interferon (IFN-γ) activity at 96 h. The serum samples were diluted 1:25 for detection. The cytokine concentrations were determined by reference to standard curves constructed with known amounts of mouse recombinant IL-2, IL-4, IL-10 or IFN-γ. The sensitivity limits of detection of IL-2, IL-4, IL-10 and IFN-γ were 16, 16, 47 and 23 pg/mL, respectively. The data from three independent experiments were analysed.

### DNA extraction and real-time PCR assay

For assessing the parasite loads in rTgPGAM 2-vaccinated mice, real-time PCR assay was used according to the published protocols [50]. Genomic DNA from the purified parasites, the liver and brain samples (100 mg each) were extracted by using a UniversalGen DNA Kit (CWBIO, China), according to the manufacturer’s instructions. The sense and antisense primer sequences of the SAG1 gene were 5′-CTGATGTCGTTCTTGCGATGTGGC-3′ and 5′-GTGAAGTGGTTCTCCGTCGGTGT-3′, respectively. PCR was performed using Applied Biosystems^®^ Real-Time PCR Instruments and SYBR Green fluorescence detection. Each reaction mixture contained 10 μL of UltraSYBR Mixture (CWBIO), 0.4 μL of each primer (20 μM), 1 μL of DNA template and 8.2 μL of sterile distilled water. Sterile water was used as a negative control, and a DNA extracted from 500 tachyzoites of the *T. gondii* RH strain was used as a positive control. All reactions were done in triplicate and the samples were incubated for 1 min at 95 °C followed by 40 cycles of 5 s at 95 °C, 15 s at 60 °C and 10 s at 72 °C.

The number of parasites in the samples was calculated from the qPCR threshold cycle (Ct) value according to a standard curve (linear curve slope: −2.8917, Y intercept: 35.8319) obtained with DNA samples from a range of serial 10-fold dilutions (5 × 10^0^–5 × 10^7^/mL) of RH strain tachyzoites under the same conditions. The tachyzoite loads in the liver and brain samples were presented as mean value of the quantity of tachyzoites estimated in per gram tissue.

### Statistical analysis

Statistical analysis was conducted with One-Way ANOVA (SPSS 13.0 software) for antibody responses, lymphocyte proliferation, cytokine assays and parasite brain and liver loads. The survival time was analysed and compared between the immunised and control groups using the Kaplan-Meier method. *P* values of less than 0.05 were considered statistically significant.

## Results

### rTgPGAM 2 elicited humoral immune responses

**Figure 1.**
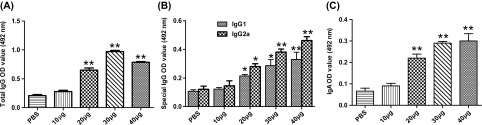
Determination of specific anti-rTgPGAM 2 humoral response in BALB/c mice. Two weeks after the immunisation schedule was completed, blood samples were obtained and antibody titres were determined by ELISA with rTgPGAM 2 as the bound target. The specific total IgG (A), IgG isotype (B) and IgA titres (C) in sera from mice vaccinated with different dosages of rTgPGAM 2 or PBS as a control were determined in duplicate. The results are expressed as the mean of the OD_492_ ± SD (*n* = 10) and are representative of three experiments. *P < 0.05, **P < 0.01 (vaccinated vs. PBS group).

To determine the specific antibody responses, blood samples were obtained and assayed using an ELISA with rTgPGAM 2 as the bound target. The IgG antibodies against rTgPGAM 2 were significantly greater in the sera of mice immunised with 20, 30 or 40 μg than those immunised with PBS and 10 μg (*P* < 0.01). In particular, 30 μg of rTgPGAM 2 provoked the stronger IgG titres (Fig. 1A). In addition, the special IgG subtypes against rTgPGAM 2 were analysed (Fig. 1B). Both IgG1 and IgG2a were found in the sera of mice vaccinated with 20, 30 or 40 μg of rTgPGAM 2, which displayed a mixed IgG1/IgG2a profile but a shift towards the Th1-type response (IgG2a/IgG1 ratio > 1). Furthermore, mice immunised with 40 μg of rTgPGAM 2 displayed higher levels of IgG2a compared with the control and other dose groups.

IgA antibodies were significantly increased in the 20, 30 and 40 μg groups compared to the PBS and 10 μg groups. The results also indicated that 30 μg of rTgPGAM 2 could elicit the highest IgA antibody response compared with the control group (*P* < 0.01). There was no difference in IgG and IgA levels between the 30 and 40 μg groups (*P* > 0.05) (Fig. 1C).

### rTgPGAM 2 induced higher titres of secretory IgA (sIgA) in mucosal washes

**Figure 2.**
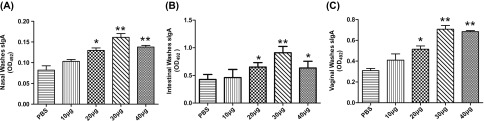
Evaluation of specific sIgA responses in mucosal washes from intranasal vaccinated mice. Groups of BALB/c mice received different doses of rTgPGAM 2 or PBS vaccine on days 0, 14 and 21. Two weeks after the last inoculation, nasal, intestinal and vaginal washes were collected to evaluate sIgA titres by ELISA. The levels of sIgA in nasal washes (A), intestinal washes (B) and vaginal washes (C) were elevated in mice nasally immunised with rTgPGAM 2 compared with vaccination with PBS. The results are expressed as the mean of the OD_492_ ± SD (*n* = 10) and representative of three experiments. *P < 0.05, ** *P* < 0.01 (vaccinated vs. PBS group).

sIgA antibodies from nasal, intestinal and vaginal washes were measured using ELISA. The levels of sIgA in the 30 μg group were significantly increased compared with those in the 10 μg and PBS groups (*P* < 0.01), although there was a significant antibody response in 20 and 40 μg groups compared with the 10 μg and control groups (*P* < 0.05) (Fig. 2). These results revealed that mucosal immune responses were elicited by rTgPGAM 2 immunisation via intranasal instillation.

### rTgPGAM 2 boosted cytokine production from spleen cells and blood samples

To characterise the immuno-modulation properties of rTgPGAM 2, the concentrations of cytokines in the supernatants of spleen cells stimulated with different doses of rTgPGAM 2 were analysed. Mice immunised with 20, 30 or 40 μg of rTgPGAM 2 displayed a significant increase in the amount of secreted IFN-γ and IL-2 as compared with the PBS and 10 μg groups. Furthermore, splenocytes of mice immunised with 30 and 40 μg of rTgPGAM 2 produced specific amounts of IL-4 (*P* < 0.05). In contrast, the IL-10 level was not changed in any group (Table 1).

**Table 1.** Cytokine production in serum samples or splenocyte cultures from immunised BALB/c mice.

Groups (*n* = 10)	Production of cytokine			
	IFN-γ	IL-2	IL-4	IL-10
splenocyte cultures (pg/mL)				
PBS	69.590 ± 5.43	861.23 ± 20.51	159.11 ± 33.21	133.88 ± 14.92
10 μg rTgPGAM 2	79.40 ± 8.14	976.28 ± 79.78	171.29 ± 26.68	134.17 ± 9.23
20 μg rTgPGAM 2	109.69 ± 3.47^☆^	1050.78 ± 86.57^☆^	181.67 ± 34.34	136.49 ± 18.46
30 μg rTgPGAM 2	187.01 ± 6.43▲	1451.35 ± 21.25▲	219.35 ± 30.26^☆^	137.28 ± 8.78
40 μg rTgPGAM 2	170.95 ± 9.21▲	1453.17 ± 99.43▲	214.81 ± 15.31^☆^	137.42 ± 10.39
serum samples (ng/mL)				
PBS	16.34 ± 2.84	27.03 ± 1.45	25.96 ± 1.84	35.29 ± 2.42
10 μg rTgPGAM 2	18.56 ± 1.28	27.56 ± 3.48	26.33 ± 1.74	37.68 ± 4.81
20 μg rTgPGAM 2	21.75 ± 1.50	29.02 ± 2.74	27.51 ± 2.89	38.34 ± 6.20
30 μg rTgPGAM 2	38.65 ± 3.43^☆^	46.94 ± 7.08^☆^	39.01 ± 2.84^☆^	38.74 ± 5.85
40 μg rTgPGAM 2	35.12 ± 3.98^☆^	43.02 ± 2.73^☆^	37.09 ± 3.01^☆^	39.49 ± 5.11

The results are presented as the arithmetic means ± standard errors of three replicate experiments. The values for IFN-γ are for 96 h. The values for IL-2 and IL-4 are for 24 h, and the values for IL-10 are for 72 h. ^☆^*P* < 0.05, ▲*P* < 0.01 (vaccinated vs. PBS group).

The amounts of cytokines in sera can reveal cellular immune responses *in vivo*. IFN-γ, IL-2 and IL-4 were significantly increased in the 30 and 40 μg groups compared with the PBS group (*P* < 0.05), whereas IL-10 was not changed in any group (Table 1). These observations are in agreement with those in the supernatants of spleen cells and indicate that immunisation with rTgPGAM 2 induced a mixed Th1/Th2 response that was orientated towards a Th1 profile.

### rTgPGAM 2 promoted cellular proliferative response

**Figure 3.**
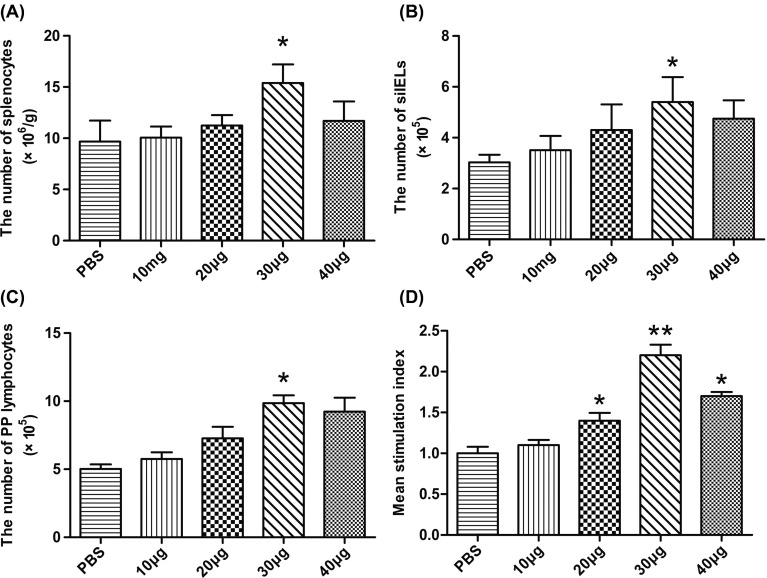
Lymphocyte proliferation responses of immunised mice. Two weeks after the last immunisation with rTgPGAM 2 or PBS, splenocytes, siIELs and PP lymphocytes were prepared and counted in a haemocytometer (A, B and C). In addition, splenocytes were cultured with ConA or rTgPGAM 2 and splenocyte proliferation (D) was measured with a CCK-8 assay. The splenocyte proliferation results are expressed as the mean stimulation index (SI) ± SD (*n* = 10) and are representative of three experiments. *P < 0.05, **P < 0.01 (vaccinated vs. PBS group).

To study the antigen-specific lymphocyte responses induced by vaccination, an *in vivo* and *in vitro* lymphocyte proliferation assay was performed. Lymphocytes from the spleen, small intestinal intraepithelial tissue and PP were isolated and counted 2 weeks after the last vaccination. The splenocyte, IEL and PP lymphocytes were significantly increased in the 30 μg rTgPGAM 2 group compared with the control group (*P* < 0.05) (Figs. 3A–3C). An *in vitro* assay was performed using 10 μg of rTgPGAM 2 as a stimulator. As shown in Figure 3d, the specific proliferative response was significantly higher in mice immunised with 30 μg of rTgPGAM 2 than in the PBS and 10 μg groups (*P* < 0.01), similar to those cells isolated from the same mice and treated with 5 μg/mL concanavalin A (Con A) as a positive control (data not shown). The lymphocyte proliferative response was also higher in mice immunised with 20 or 40 μg of rTgPGAM 2 than in the PBS and 10 μg groups (*P* < 0.05), but there was no significant difference between the 10 μg rTgPGAM 2 group and the PBS group (*P* > 0.05).

### rTgPGAM 2 reduced tachyzoite loads and increased the survival rate of *T. gondii*-infected mice

**Figure 4.**
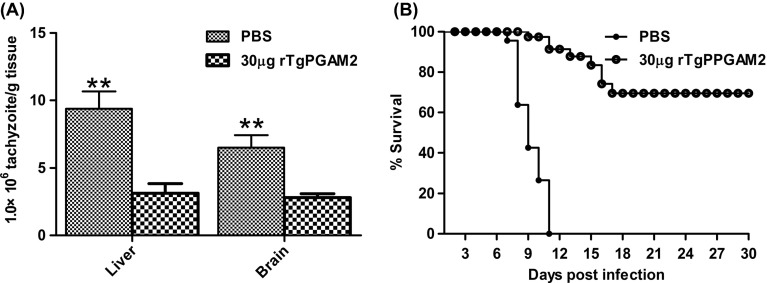
Protection of BALB/c mice against *Toxoplasma* infection. Six-week-old mice (20/group) were intranasally immunised on days 0, 14, 21 with 30 μg of rTgPGAM 2 or PBS as a control. (A) Two weeks after the last booster, the mice were challenged by gavage with 1 × 10^4^ tachyzoites of the *T. gondii* RH strain. Thirty days after the challenge, brain and liver tachyzoites were enumerated using q-PCR assay. The results are expressed as the mean ± SD (*n* = 8) and represent the numbers of tachyzoites in 1 g of tissue. **P < 0.01 (vaccinated vs. PBS group). (B) Two weeks after the last booster, the mice (12/group) were challenged with 4 × 10^4^ tachyzoites of *T. gondii* via the oral route. Survival was monitored and recorded daily for 30 days after challenge. Control mice immunised with PBS died within 11 days, and the mice immunised with rTgPGAM 2 died from days 9 to 17.

To evaluate whether rTgPGAM 2 could potentially provide protection against *T. gondii* chronic infection, the immunised mice were challenged via intragastric administration of 1 × 10^4^ *T. gondii* RH strain tachyzoites. Four weeks after the challenge, the mice were sacrificed for brain and liver tachyzoite counting. The tachyzoite loads in the brain tissues were (7.64 ± 1.47) × 10^6^/g in the control group and (2.92 ± 0.51) × 10^6^/g in the rTgPGAM 2-vacinated group. The tachyzoite loads in the liver tissues were (10.39 ± 2.17) × 10^6^/g in the control group and (3.85 ± 1.17) × 10^6^/g in the rTgPGAM 2-vacinated group. Compared with the mice in the control group, the average parasite burden was reduced significantly by 56.9% and 69.2% in the brain and liver tissues, respectively (Fig. 4A).

In addition, a lethal dosage of 4 × 10^4^ tachyzoites of the *T. gondii* RH strain was administered via an oral route. The survival time of the challenged mice was monitored and recorded one month after infection (Fig. 4B). A significant increase in the survival time and survival rate (70%) was observed in the rTgPGAM 2-immunised group compared with the control group, indicating that rTgPGAM 2 could induce partial but strong protection against *T. gondii* infection.

## Discussion

Vaccination is an appealing option to control disease and to limit the spread of the parasite either within the host or into the environment, with the expectation that most animals develop protective immune responses following primary infection with *T. gondii* [20]. In recent years, most studies have focused on the identification of vaccine candidates that can induce a protective immune response against toxoplasmosis. The soluble tachyzoite antigens and excretory secretory antigens of *Toxoplasma gondii* have been proposed as vaccine candidates against toxoplasmosis [8]. PGAMs are expressed in virtually all eukaryotic cells and in several protozoan parasites such as *Leishmania tarentolae* [42], *Trypanosoma brucei* [5] and *Entamoeba histolytica* [31]. PGAM 2 is a member of the PGAM family, TgPGAM 2 has also been identified as a soluble tachyzoite antigen, an excretory secretory antigen which can be recognised by anti-*Toxoplasma* IgM, IgG and IgA [27, 36].

In the present study, vaccination of BALB/c mice with rTgPGAM 2 elicited specific mucosal and systemic immune responses. Moreover, these specific immune responses could provide partial protection against infection by *T. gondii* RH, which was manifested by reduced tissue tachyzoites in the brain and liver of infected mice. A prolonged survival time and 70% survival rate were observed. The results demonstrate that rTgPGAM 2 is an effective candidate for the development of a vaccine against toxoplasmosis.

Cytokines play an important role in controlling *Toxoplasma gondii* dissemination in parenteral and oral infection [11]. Generally, IFN-γ induces the development of Th1 cells [28] and a Th1 response is believed to play a key role in early protective immunity when infection with *T. gondii* occurs [15]. IFN-γ can not only restrict the growth of *T. gondii* in the acute phase of the infection but also prevent reactivation of parasites from dormant cysts in the later phase: it acts as a marker of protective immunity against *T. gondii* [25, 38]. Moreover, IFN-γ and IL-2 participate in protection against parasitic invasions [35]. IL-4 is protective against development of toxoplasmic encephalitis by preventing formation of *T. gondii* cysts and proliferation of tachyzoites in the brain [39]. In our experiments, the production of IFN-γ and IL-2 in the sera and supernatant of cultured spleen cells was significantly increased in the 30 μg rTgPGAM 2 group. Meanwhile, the levels of IL-4 were also increased in rTgPGAM 2-vaccinated mice, suggesting that Th1- and Th2-type cells were generated. In addition, the increased number of spleen lymphocytes and siIELs was consistent with the increase of cytokines in different groups stimulated with rTgPGAM 2 and PBS. Thus, blood cytokines represent a valid evaluation tool for the actual response in an *in vivo* assay used to assess the immune responses of novel vaccine candidates against toxoplasmosis [32]. It would be interesting to determine the levels of these cytokines in the mucosal compartments as an additional measure of the immune responses in future studies.

Humoral immunity plays important roles in the resistance to *T. gondii*, although cell-mediated immunity plays the major role [24, 37]. Immunisation of mice with 20, 30 or 40 μg of rTgPGAM 2 resulted in the development of higher titres of total specific IgG antibodies compared with the PBS controls. A combined humoral response of both IgG1 and IgG2a was observed in the 30 and 40 μg rTgPGAM 2-vaccinated mice. The predominance of anti-rTgPGAM 2 IgG2a over IgG1 in sera from different rTgPGAM 2 dose groups also indicated that specific Th1 cells were mainly activated. The analysis of the isotype nature of the IgG response revealed high titres of anti-rTgPGAM 2 IgG1 and IgG2a antibodies in the mouse sera with the predominant IgG2a production, which is characteristic of a Th1-type response. Therefore, these results demonstrated that rTgPGAM 2 could elicit strong humoral and cellular Th1/Th2 mixed immune responses, which are essential for cell-mediated immunity and resistance against intracellular pathogens.

Secretory IgA (sIgA) secreted by the plasmocytes from the lamina propria plays a protective role against many pathogens that colonise mucosal tissues or invade the host organism by crossing mucous membranes [19, 29]. SIgA serves as a first line of defence in protecting the intestinal epithelium from enteric toxins and pathogenic microorganisms [43]. *T. gondii* naturally invades the intestine of its host and can be partially controlled by sIgA which comes from the epithelial cells of the intestine [4]. Our present results indicated that IgA antibodies in sera and sIgA antibodies in intestinal washes were significantly increased in the 30 μg rTgPGAM 2-vaccinated mice. These data were in line with those of our previous studies in which sIgA was induced by nasal immunisation with recombinant *T. gondii* Protein Disulphide Isomerase (rTgPDI) [45] and *T. gondii* actin (rTgACT) [49]. Therefore, mucosal immunisation via the nasal route has considerable potential for triggering protective immune response in all the mucosal and systemic compartments.

The lethal challenge experiments revealed that rTgPGAM 2 could prolong survival time and increase the survival rate in BALB/c mice challenged with *T. gondii* tachyzoites (RH strain). The number of brain and liver tissue tachyzoites was less than that in the control group, and the reduction rate reached 56.9% and 69.2%, respectively. This protective efficacy is greater than other recombinant protein-based vaccines such as recombinant nucleoside triphosphate hydrolase-II and recombinant actin depolymerising factor. In those studies, all immunised mice died within 9 or 14 days, respectively [18, 40]. In our previous studies, rTgPDI was measured as a candidate mucosal vaccine, but it showed a low immuno-protective potential and only increased the survival rate by approximately 31% [45].

It is well known that sIgA prevents bacterial adherence and contributes to pathogen elimination, whereas cytokines and chemokines participate in gut homeostasis as well as the recruitment of immune cells during infection [23]. Our present data revealed that rTgPGAM 2 could elicit higher levels of sIgA and cytokines in rTgPGAM 2-vaccinated mice. Therefore, we presume the invasion or proliferation of tachyzoites in the host might be suppressed by the antibodies, cytokines and immune cells that were elicited by rTgPGAM 2.

The complex life cycle of *T. gondii* includes three infectious stages, the tachyzoite, the bradyzoite and the sporozoite [10]. The rapidly dividing tachyzoite can disseminate the infection to virtually all organs and tissues of the host and can reach the foetus transplacentally. However, this important stage has scarcely been investigated as an oral infectious material because the tachyzoite is sensitive to gastric juice. It has been reported that tachyzoites can infect cats and mice via the oral route [9, 34]. In our previous studies, we have successfully established that tachyzoites infected a mouse model via the oral route [45, 47, 49]. In this model, 1 × 10^4^ tachyzoites were used to infect mice by oral gavage. Three weeks after the oral challenge, the infected mice showed rough coat, decrease in appetite and weakness/inability to obtain feed or water. Importantly, we can detect the tachyzoites in all organs and tissues of infected mice. All these phenomena show that most tachyzoites will not be killed by gastric juice and tachyzoites can infect mice via the oral route. This model will expand the recognition of tachyzoite pathogenicity and can be used for assessing the immuno-protective potential of novel candidate mucosal vaccines. It is also notable that natural infection occurs predominantly with type II strains by the ingestion of oocysts or tissue cysts [3, 16]. Therefore, future studies into the development of effective live attenuated vaccines could incorporate various protective epitopes of the type II strains, including the use of total extract antigens.

In summary, effective protection against *T. gondii* infection in mice was obtained by inoculating rTgPGAM 2 via the oral route. In the future, a multicomponent vaccine based on the recombinant forms of different parasite proteins such as rTgACT which increases the survival rate by approximately 50% [49], especially in combination with adjuvants such as cholera toxin [7], should be employed to generate better immuno-protection against *T. gondii* infection.
